# Case report: Cerebral sinus vein thrombosis in VEXAS syndrome

**DOI:** 10.3389/fmed.2024.1377768

**Published:** 2024-04-08

**Authors:** Michael Zisapel, Estelle Seyman, Jeremy Molad, Hen Hallevi, Michal Mauda-Havakuk, Tali Jonas-Kimchi, Ori Elkayam, Tali Eviatar

**Affiliations:** ^1^Rheumatology Department, Tel Aviv Sourasky Medical Center, Tel Aviv, Israel; ^2^Faculty of Medicine, Tel Aviv University, Tel Aviv, Israel; ^3^Neurology Division, Rambam Medical Center, Haifa, Israel; ^4^Department of Stroke and Neurology, Tel-Aviv Sourasky Medical Center, Tel Aviv, Israel; ^5^Department of Radiology, Tel Aviv Sourasky Medical Center, Tel Aviv, Israel

**Keywords:** VEAXS syndrome, cerebral sinus vein thrombosis, venous thromboembolism, case report, autoinflammatory disease, tocilizumab (IL-6 inhibitor)

## Abstract

VEXAS (vacuoles, E1 enzyme, X-linked, autoinflammatory, and somatic) syndrome is a newly described hemato-inflammatory acquired monogenic entity that presents in adulthood. One of the main features of VEXAS syndrome is a high venous thromboembolism (VTE) burden, with approximately 30–40% experiencing lower extremity deep vein thrombosis and a lower incidence of pulmonary embolism at approximately 10%. To date, VEXAS syndrome has not been associated with rarer forms of VTE such as cerebral sinus vein thrombosis (CSVT) and Budd–Chiari syndrome, which are well-recognized vascular manifestations in Behcet’s disease, another autoinflammatory vasculitic disease. Herein, we describe a case of acute severe extensive and fatal CSVT in a patient with VEXAS syndrome. The event occurred during a period of apparently quiescent inflammatory status, while the patient was receiving tocilizumab and a low dose of glucocorticoids. Despite treatment with anticoagulation, high-dose glucocorticoids, endovascular thrombectomy, and intracranial pressure-lowering agents, the patient suffered severe neurologic damage and ultimately succumbed to the condition 3 weeks after the onset of CSVT. To the best of our knowledge, this is the first reported case of CVST in a patient with VEXAS syndrome.

## Introduction

VEXAS syndrome is a recently recognized acquired monogenic syndrome caused by a *UBA1* mutation in myeloid cells, presenting with myelodysplastic features and diverse autoinflammatory manifestations (1). Hematologically, it is characterized by macrocytic anemia with bone marrow dysplasia, which may lead to overt myelodysplastic syndrome (MDS). The primary autoinflammatory features include fever, Sweet-like and vasculitic rashes, ear and nose chondritis, pulmonary infiltrates and venous thromboembolism (VTE), lower extremity venous thrombosis (LEVT), and pulmonary embolism (PE). However, cerebral sinus vein thrombosis (CSVT) has not been reported to date (2).

Cerebral sinus vein thrombosis is an infrequent type of VTE. Clinical presentations include intracranial hypertension characterized by headache, vomiting and visual disturbances, focal deficits, seizures, and encephalopathy. Complications include secondary brain hemorrhages and edema that may lead to herniation and death. Etiologies and associated conditions of CSVT include inherited and acquired prothrombotic conditions, including antiphospholipid syndrome, myeloproliferative neoplasm (MPN) mainly polycythemia vera (PV), head and neck infections, traumatic injuries, mechanical precipitants, nephrotic syndrome, oral contraceptives, pregnancy, and a range of inflammatory diseases (3). However, CSVT is particularly encountered in the vascular type of Behcet’s disease (BD) (4).

Behcet’s disease is a variable vessel vasculitis that can present with venous, arterial, and cardiac vasculitic-thrombotic manifestations. LEVT is the most frequent, but more unique types of VTEs such as Budd–Chiari syndrome and CSVT are widely described (4–7). While overall BD affects males and females indiscriminately, vascular BD is nine times more predominant in males (6). Another shared symptom with VEXAS is chondritis, which is a prominent feature in the MAGIC syndrome (mouth and genital ulcers with inflamed cartilage), a subtype of BD (8).

Myelodysplastic syndrome and MPNs, mainly PV, are considered a risk factor for VTE (9, 10), and CSVT has been reported as well (11). MDS may also include various inflammatory manifestations (12), which occur in up to 25% of cases. Common manifestations of “inflammatory MDS” include histiocytoid Sweet syndrome, chondritis, diverse eye inflammatory manifestations, Behcet’s-like syndrome (including gastrointestinal involvement) (13), pulmonary infiltrates, and a non-specific inflammatory syndrome. Many of those manifestations seem similar to VEXAS syndrome and prior to its recognition were attributed to inflammatory MDS and were more commonly associated with trisomy 8 chromosomal aberration (13). Nowadays, VEXAS may be considered a subgroup of “inflammatory MDS” and is indicative of severe inflammation in MDS, even though only modest dysplasia may be observed.

## Case description

In March 2023, an 82-year-old male with an established diagnosis of VEXAS presented with acute CSVT. Six years earlier, he developed left inflammatory orbital disease (IOD) accompanied by fever, weight loss, and fatigue. Over the next 2 years, he experienced recurrent alternating IOD flares, with periorbital swelling, proptosis, orbital myositis, dacryoadenitis, optic perineuritis, and posterior scleritis. Between 2018 and 2021, he had recurrent episodes of fever and one episode of a Sweet-like rash. All episodes were accompanied by macrocytic anemia, leukopenia, and elevated inflammatory markers and resolved with short courses of GCS or spontaneously. He did not develop ear or nose chondritis. Despite thorough investigations, his condition remained undiagnosed. Immune serologies including rheumatoid factor, antinuclear antibodies, C-and P-anti-neutrophil cytoplasmic antibodies, anti-proteinase 3 and anti-myeloperoxidase, anti-cardiolipin, anti beta2-glycoprotein antibodies, were negative and normal angiotensin-converting enzyme (ACE) and complement levels. Thyroid function tests were normal, but anti-thyroglobulin (404.5 U/mL, normal <150) and anti-thyroid peroxidase (107.8 U/mL, normal <75) were positive. Apart from his orbital symptoms, the other features of his disease were not felt to be compatible with an autoimmune thyroid disease. IgG4 levels were mildly elevated on two occasions (2.02–2.55 g/L, normal range 0.03–2.01) and IgG2 on another occasion 8.41 g/L (normal range 1.7–8.0). Serum protein electrophoresis and free light chain levels were without monoclonality. Bone marrow showed mild hypercellularity and dysplasia. Lacrimal gland biopsy revealed a non-specific lymphocytic infiltrate, and IgG4 staining was negative. Whole-body computed tomography (CT) scans demonstrated subpleural pulmonary infiltrates. Positron emission tomography (PET) CT revealed only diffuse FDG enhancement of bone marrow.

In May 2022, at the age of 81, he was hospitalized in an internal medicine department in our institution due to 2 weeks of fever, myalgia, and extreme fatigue. Blood tests showed worsening pancytopenia with macrocytosis with elevated inflammatory markers. Based on his medical history, a rheumatological consultation raised the suspicion of VEXAS syndrome, which was confirmed by an immediate in-hospital Sanger genetic test revealing the quite uncommon *UBA1* splice acceptor site mutation, c.118–1 G > C, variant allele fraction of 50%. He was diagnosed with VEXAS syndrome. Ultrasound Doppler and chest CT with contrast performed at the time of diagnosis demonstrated neither LEVT nor PE. Treatment with 40 mg of prednisone was initiated but when tapered to 20 mg, his fever and fatigue recurred. Subcutaneous tocilizumab (162 mg once a week) was administered with the resolution of fever. IOD flares and rash did not reoccur. Hemoglobin and inflammatory markers normalized. Prednisone was gradually tapered to 7.5 mg. Under this steady immunosuppressive regimen for 4 months, he felt well and his blood tests were stable, suggesting that his disease was quiescent.

**Figure 1:**
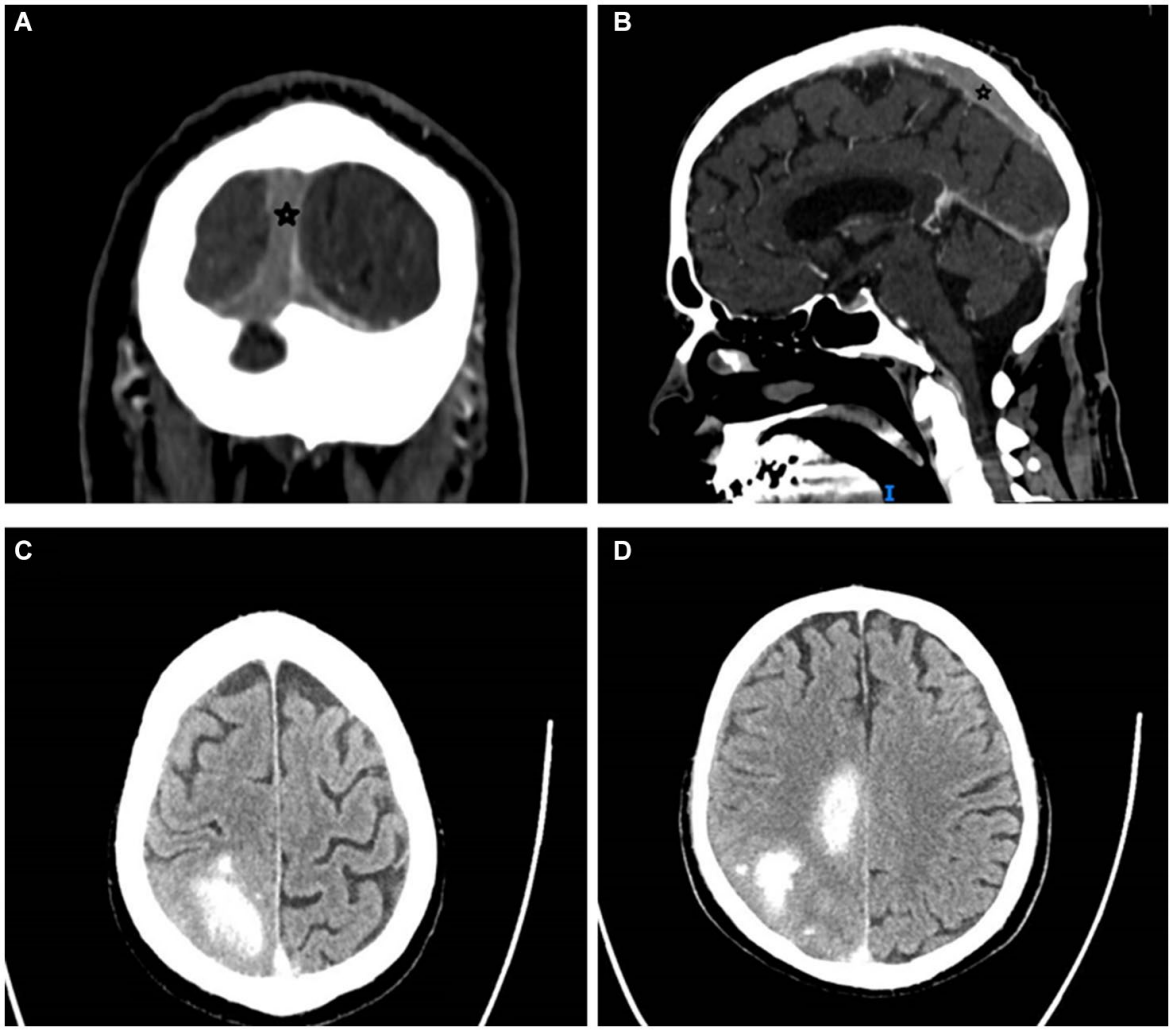
Cerebral sinus vein thrombosis at presentation. Star mark signifies sinus vein thrombosis. **(A)** Occluded sagittal sinus, coronal view, CT venography. **(B)** Occluded superior sagittal sinus, sagittal view, CT venography. **(C,D)** Right parenchymal hemorrhages and subarachnoid hemorrhage, transverse view, CT without contrast. CT, computed tomography.

In March 2023, the patient developed a few days of weakness followed by sudden walking instability, confusion, and left-sided neglect without any complaints of headache, nausea, or vomiting. CT scan with CT venography in the emergency department demonstrated CSVT of superior sagittal, left and right transverse, right sigmoid sinuses and right jugular vein, and secondary right hemisphere parenchymal and subarachnoid hemorrhages, along with brain edema and compression signs (Figure 1).

**Figure 2:**
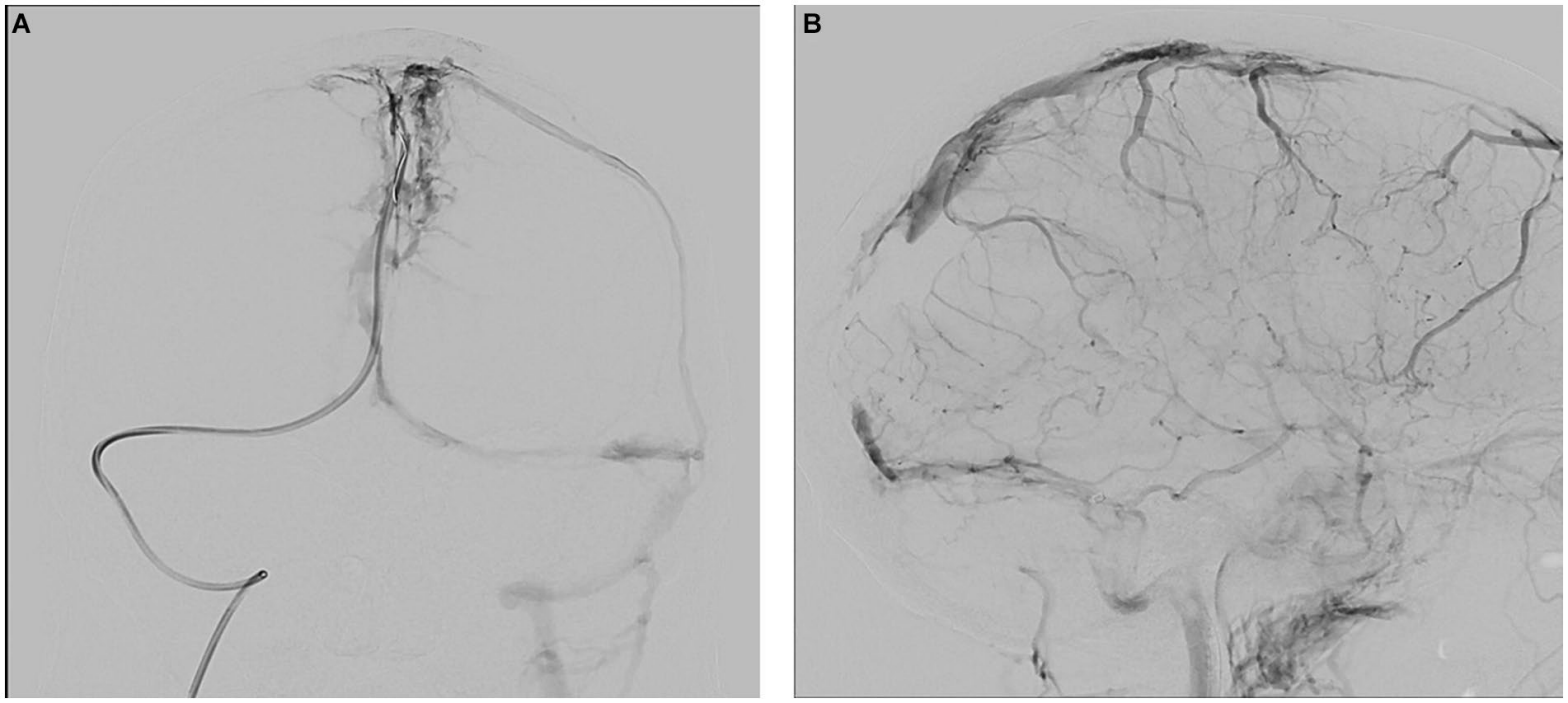
Therapeutic angiography (venous phase) at presentation. **(A)** Occluded superior sagittal sinus, coronal view. **(B)** Occluded sagittal, right sigmoid and right transverse sinuses, sagittal view.

Upon arrival, the patient’s blood tests showed hemoglobin of 13 mg/dL, borderline leukocyte count, and mild thrombocytopenia. Inflammatory markers under tocilizumab were normal. The patient’s condition rapidly deteriorated, and he developed seizures and decreased consciousness, requiring intubation. He was treated with high-dose intravenous methylprednisolone, full parenteral anticoagulation (AC), antiepileptics, endovascular thrombectomy, and intracranial pressure (ICP) lowering agents (Figure 2).

Nevertheless, he suffered extensive neurologic damage (left-side hemiplegia and facialis, right-leg paresis, left hemianopsia, anarthria, and dysphagia). Three weeks after his presentation, he passed away due to massive aspiration.

**Figure 3:**
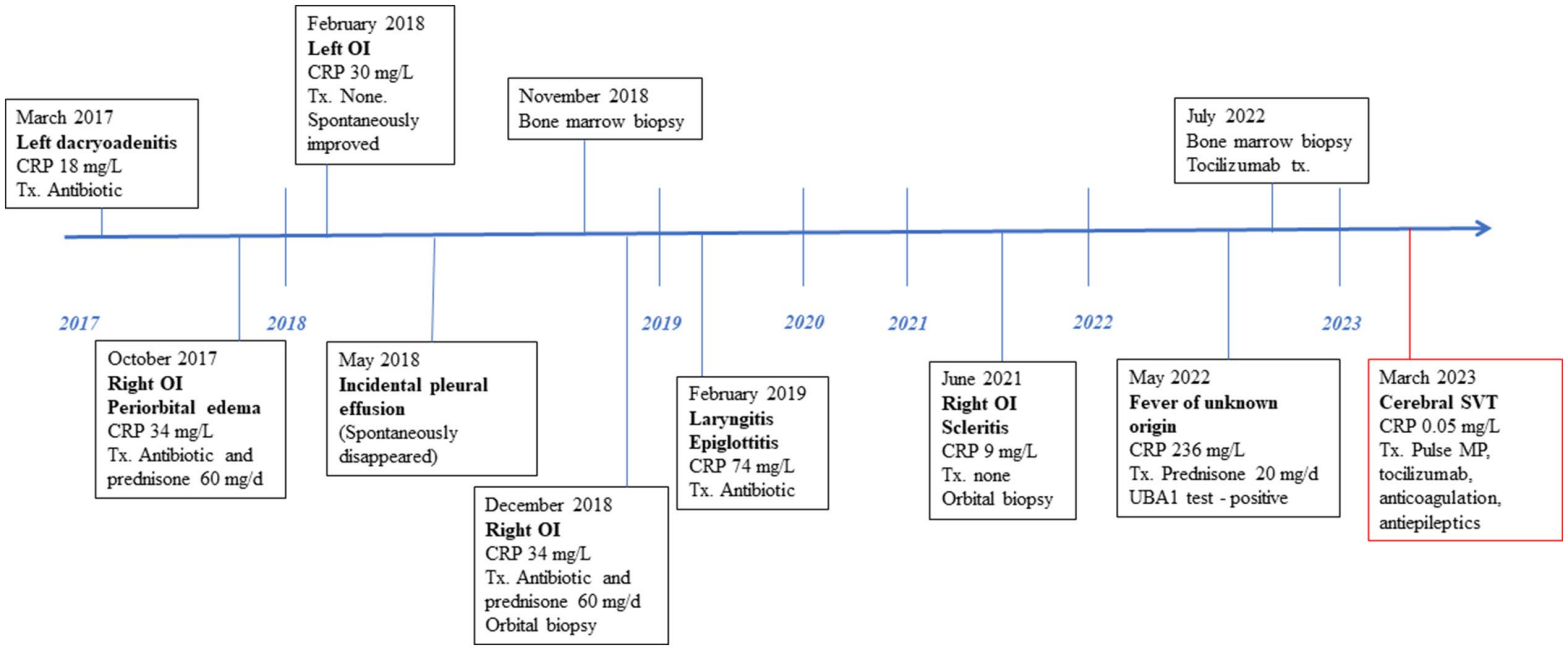
Clinical and therapeutic timeline. CRP, C-reactive protein; Tx, treatment; OI, ocular inflammation; MP, methylprednisolone; SVT, sinus vein thrombosis.

See Figure 3 for the patient timeline.

## Discussion

We presented a case of a VEXAS patient who developed severe CSVT despite immunosuppressive treatment (IS) with low-dose GCS and tocilizumab while apparently in a quiescent inflammatory status and without prior VTE history. Unfortunately, the patient had several CSVT poor prognostic factors; male sex, old age, deep sinus involvement, right hemisphere brain hemorrhage, brain edema and compression, and severely altered mental status (14). Malignancy is also a poor prognostic factor in CSVT, and VEXAS might be considered a hematologic neoplasm.

VEXAS is considered a preclinical and sometimes an overt inflammatory MDS, and shares clinical similarities with autoinflammatory and vasculitic conditions, specifically relapsing polychondritis (RP) and BD (skin and ocular manifestations) including vascular BD (LEVT, PE). Inflammatory MDS, as VEXAS is, has been associated with a Behcet’s-like syndrome (13). VTE risk is increased in VEXAS, BD, and MDS itself (including CSVT in the latter two). VEXAS, an X-linked genetic condition, almost solely affects males, and similarly, vascular BD is highly more prevalent in males.

A clinical interface exists between inflammatory MDS, VEXAS, RP, and BD-like manifestations. VEXAS is a unifying representation of these, which is not surprising, given the shared pathogenesis involving innate immunity of myeloid-driven inflammation and inflammasome activation (1, 10, 15).

In BD, VTEs are considered to be inflammatory in nature, rather than purely thrombotic, and therefore, IS therapy is more important than AC, long-term AC is not formally recommended, and prophylactic AC is not customary (5–7). The same logic may be applied to VEAXS, but as seen in our case, severe VTE developed despite IS and during an allegedly quite inflammatory status. In our patient, inflammation may have been masked by tocilizumab, preventing fever and normalizing inflammatory markers, while covertly developed and exploded as a catastrophic VTE event. This raises the dilemma of whether prophylactic AC should be given to VEXAS patients without a prior VTE history while considering their old age and bleeding risks. Eventually, if VEXAS truly holds Behcet’s-like vascular complications, the theoretical possibility of pulmonary artery aneurysm and its bleeding risk should not be disregarded.

Ruxolitinib is a Janus kinase (JAK)-1,2 inhibitor used to treat MPNs and has also been tried with some success in VEXAS patients (16). However, JAK inhibitors have been associated with an increased risk of VTEs (17), specifically in rheumatoid arthritis patients (18), although data are not consistent (19). Nevertheless, in PV and myelofibrosis, ruxolitinib has shown evidence of decreasing thrombotic events (20). Therefore, ruxolitinib may be possibly a potential treatment option for both inflammatory and thrombotic manifestations in VEXAS patients.

Our patient carried the less common splice acceptor site mutation, which is present in 7% of patients according to the French register (21). Data regarding clinical characteristics are scarce, but patients are significantly older and display more ocular involvement, as was the case in our patient. Similar to patients with the Valine mutation, patients with splice site mutations more frequently exhibit constitutional symptoms, lung involvement, and overt MDS, when compared to those with the leucine and threonine mutations. Interestingly, splice site mutation patients also have a significantly higher gastrointestinal (GI) involvement than other genotypes (37.5% vs. 12%) (21). This corresponds with the frequent GI involvement described in the past in Behcet’s-like MDS patients (13). Regarding the incidence of VTE, no significant differences were observed between patients with splice site mutations and other genotypes (25% vs. 36%), although the splice site mutations are less frequent and hence less described, and therefore, follow-up duration of patients is shorter. Pathophysiologically, similar to the canonical M41 mutations, the splice site mutations are responsible for a shift from the cytoplasmic UBA1b isoform to the catalytically abnormal UBA1c isoform (15, 22).

To conclude, we presented the first case of CSVT in a VEXAS patient, highlighting the potential for devastating vascular manifestation in this syndrome. Currently, there are insufficient data to support prophylactic AC in VEXAS. However as our understanding of the spectrum of VEXAS manifestations, complications, genotype–VTE risk association, and treatment approach, is evolving, specific immunomodulators and AC may be recommended in future.

## Data availability statement

The original contributions presented in the study are included in the article/supplementary material, further inquiries can be directed to the corresponding author.

## Ethics statement

Ethical approval was not required for the study involving humans in accordance with the local legislation and institutional requirements. Written informed consent to participate in this study was not required from the participants or the participants' legal guardians/next of kin in accordance with the national legislation and the institutional requirements. Written informed consent was not obtained from the individual(s) for the publication of any potentially identifiable images or data included in this article because the patient is deceased, his next of kin (a child) gave informed consent.

## Author contributions

MZ: Conceptualization, Methodology, Writing – original draft, Writing – review & editing, Investigation. ES: Writing – review & editing. JM: Writing – review & editing. HH: Writing – review & editing. MM-H: Writing – review & editing. TJ-K: Visualization, Writing – review & editing. OE: Methodology, Supervision, Writing – original draft, Writing – review & editing. TE: Conceptualization, Methodology, Supervision, Visualization, Writing – original draft, Writing – review & editing.
